# THE TRUTH ABOUT AGE DIFFERENCES IN PERFORMANCE ON VARYING LENGTHS OF LIKERT-TYPE TEST ITEMS

**DOI:** 10.1093/geroni/igz038.2598

**Published:** 2019-11-08

**Authors:** Julie Lutz, Aaron Metzger, Nicholas A Turiano, Rachael Spalding, Emma Katz, Barry Edelstein

**Affiliations:** West Virginia University, Morgantown, West Virginia, United States

## Abstract

Guidelines for self-report assessment with older adults emphasize the use of shorter Likert-type or agree/disagree response formats to reduce cognitive load (e.g., Yesavage et al., 1983). However, these suggestions are not founded on empirical studies directly comparing younger and older adults’ responses on different scales. Thus, the current study tested differential responding on varying Likert-type response scale lengths between younger, middle-aged, and older adults. Participants completed three versions of the International Personality Item Pool (IPIP) Neuroticism scale with 3, 5, and 7 Likert-type response scale lengths in counterbalanced orders with other questionnaires between versions. Six multi-group confirmatory factor analyses (CFAs) assessed measurement invariance across scale lengths and age groups. Invariance of convergent validity networks was also assessed with multi-group CFAs of the associations between the IPIP and measures of depression, anxiety, anger, worry, and affect. The final sample consisted of 835 adults (327 18-44; 279 45-64; and 229 65 or older) via Amazon Mechanical Turk. Measurement invariance was supported in analyses by age within each scale length and by scale length within each age group, indicating that response patterns across all scale lengths and age groups did not significantly differ. Analyses of convergent validity also supported invariance, suggesting that responses across all scale lengths and age groups reflect the same underlying construct. This study indicates that, among community-dwelling adults, shortened response scale lengths do not yield significantly different or more valid responses for older adults compared to younger adults.

